# A rare case of a combination of ectopic kidney and medial arcuate ligament syndrome: a case report

**DOI:** 10.1186/s12894-023-01361-z

**Published:** 2023-11-18

**Authors:** Chun-Kai Hsu, Wen-Tsang Hsu, Wan-Ling Young, Shu-Yu Wu

**Affiliations:** 1 Department of Urology, Taipei Tzu Chi Hospital, Buddhist Tzu Chi Medical Foundation, No. 289, Jianguo Rd., Xindian Dist, New Taipei City, 231 Taiwan; 2 Department of Urology, School of Medicine, Tzu Chi University, Hualien, Taiwan; 3 Department of Urology, Keelung Hospital, Ministry of Health & Welfare, Keelung, Taiwan

**Keywords:** Median arcuate ligament syndrome, Celiac artery compression syndrome, Ectopic kidney, Pelvic kidney

## Abstract

**Background:**

Ectopic kidney and median arcuate ligament syndrome are both rare conditions. The clinical presentation and diagnosis of these conditions are not well studied. There are no reports on the combination of these two rare conditions.

**Case presentation:**

We report a 24-year-old woman with fever, dysuria, urinary frequency and left flank pain for two days. The primary diagnoses in the clinic were left acute pyelonephritis and left hydronephrosis due to throbbing pain in the left costovertebral angle and pyuria. However, further computed tomography showed right ectopic pelvic kidney, left renal pelvis dilatation without definite ureteral lesion, good bilateral renal contrast enhancement, and compression of the celiac axis due to obstruction by the median arcuate ligament. Chronic abdominal symptoms were reported by the patient after repeat history taking. The patient’s condition was fully explained and discussed with her and her family, but they refused further therapy. After the acute pyelonephritis began improving, the patient was discharged for follow-up at our outpatient clinic.

**Conclusion:**

We present an extremely rare case of a combination of two rare conditions: ectopic kidney and median arcuate ligament syndrome. No study to date has reported on the relationship between the two diseases. Given the rarity of the two conditions, no evidence or even a hypothesis exists to explain the possible etiology of their combination. More reports are required to enhance the understanding of these rare conditions.

## Background

Ectopic kidney and median arcuate ligament (MAL) syndrome are both rare conditions. The incidence of pelvic kidney is about 3-4.5% in previous report [1].Most ectopic kidneys are found incidentally on radiographic examination for atypical abdominal symptoms [2]. The possible problems after ectopic kidney include structural and architectural anomalies in the kidney itself, abnormal pelvic vascular pattern, and the relationship with other pelvic organs [3]. Following the anatomical abnormality, conditions such as ureter obstruction, infection, and urolithiasis may occur. Patients should have regular clinic follow-ups and be alert to any abdominal or pelvic symptoms.

MAL syndrome, also called celiac artery compression syndrome, is a rare condition resulting from anatomical compression of the celiac axis or celiac ganglion by the MAL and diaphragmatic crura [4]. The incidence of MAL syndrome in the general population is not well understood. It is more commonly reported in women aged 30 to 50 years or with a thin body habitus [5]. The clinical symptoms are often non-specific gastrointestinal symptoms, including abdominal pain, postprandial abdominal pain, nausea and vomiting, weight loss, and bloating [6]. For such a rare disease, it is commonly a diagnosis of exclusion or an incidental finding during image analysis for other reasons. We report the case of a young woman presenting with both these rare conditions. This report follows the CARE checklists for case reporting.

## Case presentation

The patient was a 24-year-old female who visited our hospital due to fever, dysuria, urinary frequency and left flank pain for two days. She had no systemic disease or a family history of systemic disease and seldom visited a hospital. The primary survey at our urology clinic included physical examinations, urinalysis, renal and bladder sonography, and abdominal X-ray according to her symptoms. Her abdomen was soft and flat, with no palpable mass. A throbbing pain was noted during the examination of the left costovertebral angle. The urinalysis showed pyuria and hematuria. Abdominal X-ray showed no urolithiasis. However, renal sonography showed dilatation of the left renal pelvis and an absent right kidney. The right kidney was found in the pelvic cavity near the bladder by bladder sonography. The primary diagnoses were left acute pyelonephritis, left hydronephrosis, and right ectopic kidney. The patient was admitted to our hospital for further treatment and evaluations due to acute illness.

**Fig. 1:**
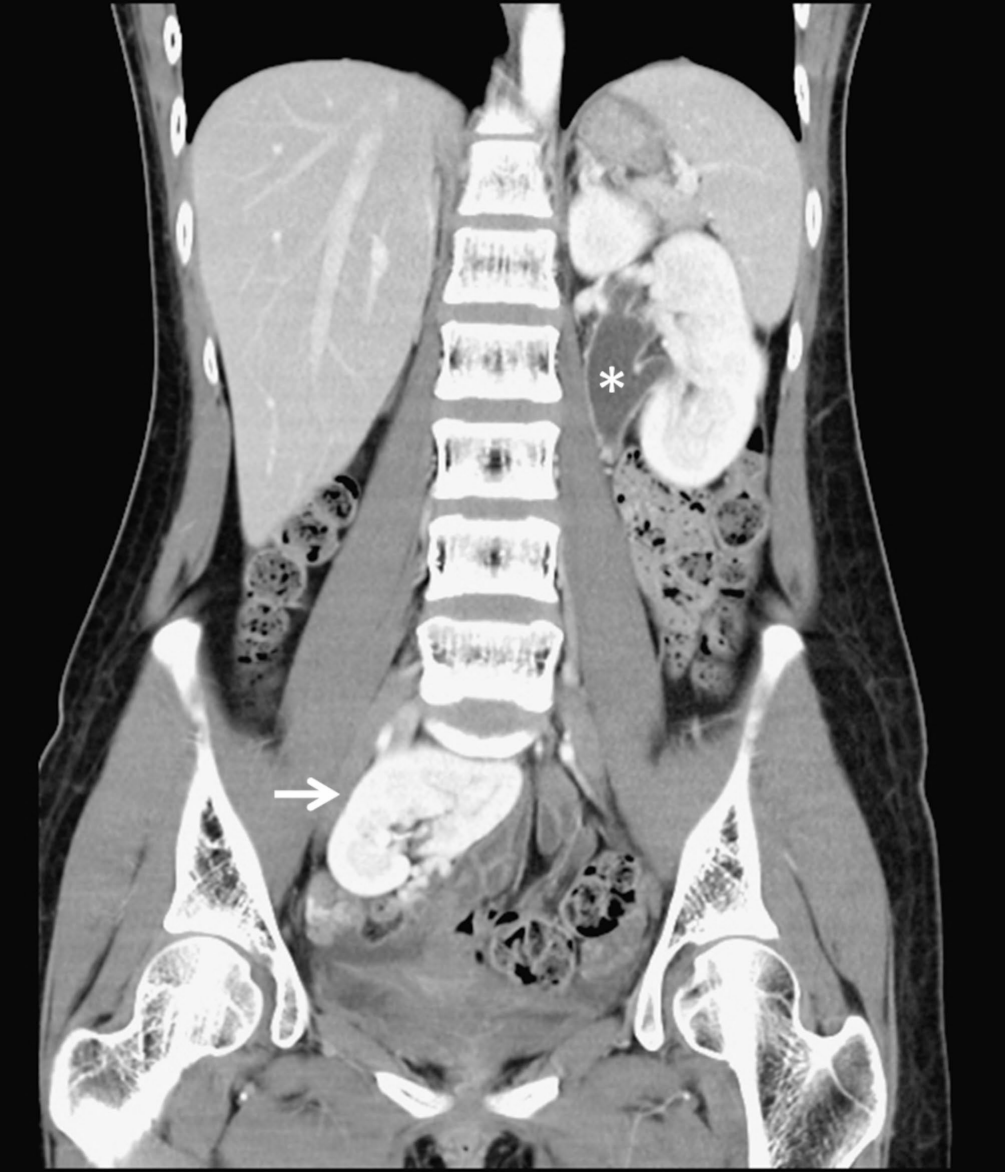
Abdomen-to-pelvis computed tomography with contrast showing the right ectopic pelvic kidney (arrow) and left dilated renal pelvis (✽)

**Fig. 2:**
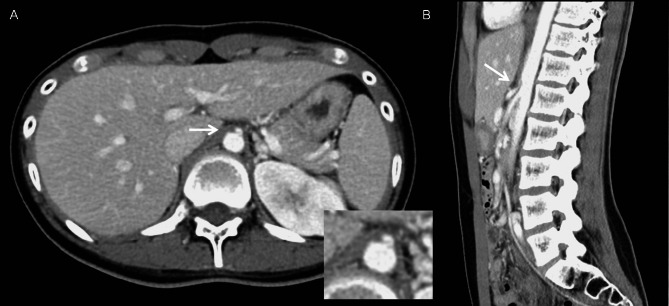
Abdomen-to-pelvis computed tomography with contrast showing the thickened median arcuate ligament and diaphragmatic crura (**A**, arrow) and the kinked proximal celiac artery, with a hooked appearance, and post-stenotic dilatation (**B**, arrow)

After admission, the initial vital signs indicated fever (38.2℃), tachycardia (heart rate 124/min), normal respiratory rate (16/min), and low blood pressure (101/57mmHg). Laboratory data showed a white blood cell count of 8390/µL, hemoglobin level of 11.9 g/dL, and BUN/CRE of 10.4/0.59. Broad-spectrum empirical antibiotics were given for infection control. Computed tomography (CT) was performed to identify the etiology of the left hydronephrosis. The CT showed right ectopic kidney in the pelvis (Fig. 1), dilatation of left renal pelvis without definite ureteral lesion (Fig. 1), and good bilateral renal contrast enhancement. In addition, compression of the celiac axis due to obstruction by the MAL was incidentally noted (Fig. 2). We rechecked her history for abdominal symptoms due to the CT images. Some evidence was found during repeat history taking. She was underweight (158 cm, 42 kg, BMI = 16.82) and had experienced postprandial nausea, abdominal pain, and delayed gastric emptying for many years.

The findings were explained to the patient and her family. After a full explanation and discussion, they refused further evaluations and treatments for the celiac artery obstruction due to worry about side effects. Because there was no obvious ureteral obstruction on imaging, drainage treatment was not initially recommended for the patient. Thereafter, no further surgical intervention was scheduled due to significant improvement in clinical symptoms after conservative treatment. After three days of antibiotic use (cefazolin, 1 g, intravenous drip, every 8 h), her symptoms improved. The only other symptomatic treatment is acetaminophen. The urine culture showed *Escherichia coli* without drug resistance. Therefore, she was discharged for follow-up at our outpatient clinic with oral antibiotics (cephalexin, 500 mg/cap, four times a day). At clinic visit after one week antibiotic treatment, urinalysis was normal. At the same time, the patient did not have any symptoms.

## Discussion and conclusions

The human kidney continues to develop between the sixth and eighth week of life, and the kidneys ascend during the ninth week. As the kidney travels cephalad, the vascular supply arises from successive transient aortic offshoots. The failure of kidney ascension is caused by fetal renal blood supply degeneration or lack of factors responsible for ureteral growth and elongation [7, 8]. The exact reasons for the failure of the kidney to ascend remain unclear. Hypotheses include abnormalities of the ureteral bud and metanephric blastema, genetic variants, teratogenic effects, and anomalous vasculature physically blocking ascent [9]. A kidney showing incomplete renal ascent will reside in an ectopic position where its ascent stopped. A kidney that does not ascend at all remains in the pelvis and becomes a pelvic kidney. It usually lies opposite the sacrum and caudal to the aortic bifurcation. Due to their failure to ascend, pelvic ectopic kidneys remain unrotated and often have a fetal blood supply from the iliac vessels or from the distal aorta [3].

Some diseases that are reported to be associated with pelvic ectopic kidney include urolithiasis, ureteropelvic junction (UPJ) obstruction, and extrarenal calices [10, 11]. These possible complications are hypothesized based on evidence of delayed emptying of the renal pelvis caused by UPJ obstruction secondary to its abnormal anatomy from studies on horseshoe kidney [12]. However, currently there is no reported evidence of incidence of stones in pelvic kidneys. Renal tumor is another potential issue in those with developmental or anatomical abnormalities. However, to date, only a few case reports have mentioned malignancy in ectopic pelvic kidney. There is no evident increase in the risk of malignant change in ectopic pelvic kidneys [3].

The common symptoms include abdominal pain, fever, hematuria or incontinence from an ectopic ureter or small ectopic pelvic kidney. An abdominal mass may be palpable during physical examinations. However, past case series suggest that only a small proportion of patients develop symptoms (20.2%) [13]. For women of childbearing age, the risks of pregnancy and childbirth should be considered. However, there are currently no reports had focused on the risks of ectopic kidneys in childbirth. Intuitively, anatomic abnormalities may affect the safety of caesarean section. Compared with the rare ectopic kidney, what can be used as a comparison is the risk of childbirth after kidney transplantation. The following complications in pregnant transplant recipients are higher than in the general population in previous reports: stillbirth rate, ectopic pregnancies, preeclampsia, pregnancy-induced hypertension, gestational diabetes, cesarean section delivery, preterm birth and low birth weight [14]. However, apart from the same location of the kidneys (ectopic kidney and graft kidney), there are huge differences between the two in other pathophysiological aspects. Further research is still needed to confirm the relationship.

MAL syndrome has been known for more than one hundred years. Due to its rare and variable symptoms, it remains a controversial diagnosis. One commonly accepted theory suggests that increased demand for blood flow through a compressed celiac artery leads to foregut ischemia resulting in epigastric pain, although the development of collateral vessels usually prevents severe ischemia. Another hypothesis is that the symptoms are due to a combination of chronic compression and over-stimulation of the celiac ganglion, which leads to direct sympathetic pain and/or splanchnic vasoconstriction and ischemia [4]. In addition, vascular steal of blood flow by large collateral vessels may lead to symptoms of celiac artery compression in individuals with an occluded celiac trunk [15].

The symptoms of MAL syndrome are usually non-specific and may be caused by most other abdominal disorders. Abdominal pain, unintentional weight loss, delayed gastric emptying; weight loss, nausea, and vomiting are reported [6, 16]. Physical examination is usually normal. Patients may undergo many examinations to exclude other etiologies before the final diagnosis of MAL syndrome is made. Duplex abdominal ultrasonography could be the primary tool to identify MAL syndrome. The celiac axis tracks cephalad during deep expiration, which will lead to aggravated external compression and elevated velocities with post-stenotic dilatation [17, 18]. CT angiography and magnetic resonance angiography are other noninvasive imaging techniques that can be used in the diagnosis of MAL syndrome. They provide three-dimensional reconstruction images that allow direct visualization of the compressed artery from different angles. For those who plan surgical intervention to release the compression, three-dimensional serial images are beneficial to both preoperative and postoperative evaluations [19].

The aims of MAL syndrome treatment are to relieve the compression, retrieve adequate blood flow, and finally resolve the chronic symptoms. Surgical interventions are required to cure this condition and could be performed as either open or laparoscopic surgery for decompression and reconstruction. First, the MAL and the diaphragmatic crura are decompressed away from the celiac artery. Then, vascular reconstruction may be needed to provide adequate blood flow [20, 21]. Limited evidence suggests that endovascular intervention is an unsuccessful first-line therapy. However, the procedure is useful for those with residual symptoms or stenosis after operative interventions [6]. Jimenez et al. [22] published a large review on the treatment of MAL syndrome and they found that laparoscopic or open ligament release, celiac ganglionectomy, and celiac artery revascularization can provide sustained symptom relief in most patients, even with very high conversion rates. On the other hand, the role of arterial revascularization remains unclear.

In addition to the above abnormalities, vesicoureteral reflux (VUR) may also be one of the causes of left hydronephrosis. However, we did not perform further studies such as DMSA scans. The main reason is that our hospital lacks nuclear medical examination capabilities and the patient is unwilling to transfer to another hospital for examination and treatment. So the urinary tract infection was treated with medication, and the symptoms improved smoothly. Vesicoureteral reflux (VUR) is defined as retrograde regurgitation of urine from the urinary bladder up the ureter and into the collecting system of the kidneys. In high grade VUR (involving kidney), ureter dilatation or tortuous is common, which was not presented in our patient. Therefore, it seems that this patient does not likely to have VUR. We tend to think that temporary hydronephrosis is the result rather than the cause of acute infection. However, we cannot totally exclude the possibility of VUR in the lack of voiding cystourethrography.

We present an extremely rare case with two rare conditions: ectopic kidney and MAL syndrome. No study to date has reported on the relationship between the two conditions. Not enough evidence or even a hypothesis exists to explain the etiology of this rare combination of conditions. We believe that the co-occurrence of the conditions might be a coincidence. More reports are required to enhance the understanding of these rare conditions.

## Data Availability

All data are available on request of the corresponding author.

## Abbreviations

MAL: Median arcuate ligament
CT: Computed tomography
UPJ: Ureteropelvic junction
